# Active School Transportation in Winter Conditions: Biking Together is Warmer

**DOI:** 10.3390/ijerph17051524

**Published:** 2020-02-27

**Authors:** Anna-Karin Lindqvist, Marie Löf, Anna Ek, Stina Rutberg

**Affiliations:** 1 Department of Health Sciences, Luleå University of Technology, 971 87 Luleå, Sweden; stina.rutberg@ltu.se; 2 Department of Biosciences and Nutrition, Karolinska Institutet, 141 83 Huddinge, Sweden; marie.lof@ki.se (M.L.); anna.ek@ki.se (A.E.); 3 Department of Medical and Health Sciences, Linköping University, 581 85 Linköping, Sweden

We wish to update Section 2.3 (Data Collection) in our paper published in the *International Journal of Environmental Research and Public Health* (*IJERPH*) [1]. In the beginning of the first paragraph in the data collection section, we would like to add the following sentence: With inspiration from an unpublished manuscript by Lögdberg et al., the photovoice method was developed through discussions in the research team, and through a review of relevant literature [2,3]. The changes do not affect the results. The manuscript will be updated and the original will remain online on the article webpage with a reference to this addendum.
